# 1-[1-(Hydroxy­imino)eth­yl]-*N*-(2-methoxy­phen­yl)cyclo­propane­carboxamide

**DOI:** 10.1107/S1600536809022260

**Published:** 2009-06-20

**Authors:** Jun-Ling Wang, Shuang-Ming Meng, Mao-Zhong Tian, Feng Feng

**Affiliations:** a College of Chemical Engineering, Shanxi Datong University, Datong 037009, People’s Republic of China

## Abstract

The title compound, C_13_H_16_N_2_O_3_, adopts an *E* configuration with respect to the C=N bond and an intra­molecular N—H⋯N hydrogen bond results in the formation of a six-membered ring. In the crystal, inter­molecular O—H⋯O hydrogen bonds link the mol­ecules into a chain propagating along the *b* axis. Very weak π–π stacking inter­actions [centroid–centroid distance = 4.18 (2) Å] may further consolidate the packing, forming a two-dimensional supra­molecular network.

## Related literature

For background to cyclo­propane derivatives, see: Liu & Montgomery (2006 ▶); Ogoshi *et al.* (2006 ▶).
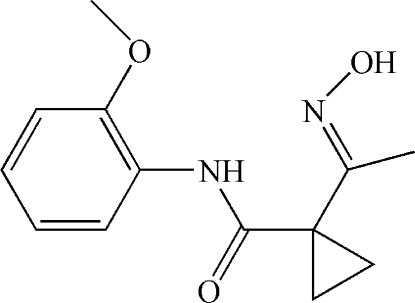


## Experimental

### 

#### Crystal data

C_13_H_16_N_2_O_3_
*M* *_r_* = 248.28
Monoclinic, 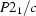

*a* = 16.062 (6) Å
*b* = 5.483 (2) Å
*c* = 14.250 (6) Å
β = 100.055 (6)°
*V* = 1235.7 (8) Å^3^
*Z* = 4
Mo *K*α radiation
μ = 0.10 mm^−1^
*T* = 293 K
0.41 × 0.29 × 0.20 mm

#### Data collection

Bruker SMART APEX CCD diffractometer
Absorption correction: multi-scan (*SADABS*; Bruker, 1999 ▶) *T*_min_ = 0.96, *T*_max_ = 0.99
6430 measured reflections
2432 independent reflections
1520 reflections with *I* > 2σ(*I*)
*R*_int_ = 0.044

#### Refinement

*R*[*F* ^2^ > 2σ(*F* ^2^)] = 0.073
*wR*(*F* ^2^) = 0.170
*S* = 1.09
2432 reflections
169 parameters
2 restraints
H atoms treated by a mixture of independent and constrained refinement
Δρ_max_ = 0.21 e Å^−3^
Δρ_min_ = −0.20 e Å^−3^

### 

Data collection: *SMART* (Bruker, 1999 ▶); cell refinement: *SAINT* (Bruker, 1999 ▶); data reduction: *SAINT*; program(s) used to solve structure: *SHELXS97* (Sheldrick, 2008 ▶); program(s) used to refine structure: *SHELXL97* (Sheldrick, 1008); molecular graphics: *SHELXTL-Plus* (Sheldrick, 2008 ▶); software used to prepare material for publication: *SHELXL97*.

## Supplementary Material

Crystal structure: contains datablocks global, I. DOI: 10.1107/S1600536809022260/hb2995sup1.cif

Structure factors: contains datablocks I. DOI: 10.1107/S1600536809022260/hb2995Isup2.hkl

Additional supplementary materials:  crystallographic information; 3D view; checkCIF report

## Figures and Tables

**Table 1:** Hydrogen-bond geometry (Å, °)

*D*—H⋯*A*	*D*—H	H⋯*A*	*D*⋯*A*	*D*—H⋯*A*
N1—H1*N*⋯N2	0.856 (17)	1.94 (2)	2.670 (3)	142 (3)
O3—H3*O*⋯O2^i^	0.85 (4)	1.93 (2)	2.751 (3)	162 (4)

Symmetry code: (i) 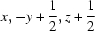.

**Fig. 1.**
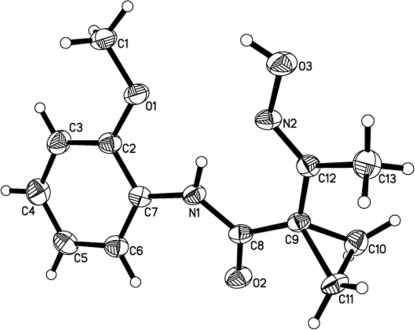
Molecule structure of (I) with displacement ellipsoids drawn at the 30% probability level for non-H atoms.

**Fig. 2.**
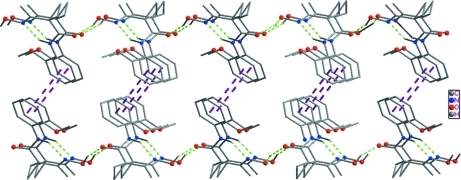
View of the two-dimensional supramolecular structure of (I): hydrogen bonds and π-π interactions are shown as dashed lines.

## supplementary crystallographic information

### Comment

Cyclopropane and their derivatives are a significant class of compounds which can be used in a variety of studies such as organic synthesis, catalytic reaction and so on (Liu & Montgomery, 2006; Ogoshi *et al.*, 2006). In order to extend our work on structural characterization of cyclopropane compounds, we report the synthesis and the X-ray structure of the title compound, (I), in this paper (Fig. 1).

The title molecule adopts an E configuration with respect to C=N bond. There is an intramolecular O—H···N hydrogen bonds, forming of a six-membered ring (Table 1) and the intermolecular O—H···O hydrogen bonds link the molecules into a one-dimensional chain along the *b* axis. The crystal structure is further stabilized by π-π interaction involving the benzene rings: *Cg*1···*Cg*1 (1 - *x*, 1 - *y*, 1 - *z*) = 4.18 (2) Å, where *Cg*1 denotes the centroid of the C2—C7 (Fig. 2).

### Experimental

To a solution of 1-acetyl-*N*-(2-methoxyphenyl)cyclopropanecarboxamide (2.33 g, 10 mmol) and NaOAc (1.64 g, 20 mmol) in EtOH (25 ml) and H_2_O (1 ml) was added NH_2_OH.HCl (1.39 g, 20 mmol) in one portion. The reaction mixture was stirred at room temperature for 12 h, and then poured into ice-water (200 ml) under stirring.  A white solid was precipitated, which was filtered and the residue was purified by a flash silica gel column chromatography to give colourless blocks of (I) (eluent: ether/ethyl acetate = 1/3 v/v).

### Refinement

The N- and O-bound H atoms were located in a difference map and their positions were freely refined. The C-bound H atoms were geometrically placed (C—H = 0.93–0.97Å) and refined as riding.  The constraints *U*_iso_ = 1.2*U*eq(C,N) or 1.5*U*eq(methyl C,O) were applied.

### Crystal data

**Table:**

C_13_H_16_N_2_O_3_	*F*(000) = 528
*M**_r_* = 248.28	*D*_x_ = 1.335 Mg m^−^^3^
Monoclinic, *P*2_1_/*c*	Mo *K*α radiation, λ = 0.71069 Å
Hall symbol: -P 2ybc	Cell parameters from 2432 reflections
*a* = 16.062 (6) Å	θ = 1.3–26.1°
*b* = 5.483 (2) Å	µ = 0.10 mm^−^^1^
*c* = 14.250 (6) Å	*T* = 293 K
β = 100.055 (6)°	Block, colourless
*V* = 1235.7 (8) Å^3^	0.41 × 0.29 × 0.20 mm
*Z* = 4	

### Data collection

**Table:**

Bruker SMART APEX CCD diffractometer	2432 independent reflections
Radiation source: fine-focus sealed tube	1520 reflections with *I* > 2σ(*I*)
graphite	*R*_int_ = 0.044
ω scans	θ_max_ = 26.1°, θ_min_ = 1.3°
Absorption correction: multi-scan (*SADABS*; Bruker, 1999)	*h* = −9→19
*T*_min_ = 0.96, *T*_max_ = 0.99	*k* = −6→6
6430 measured reflections	*l* = −17→17

### Refinement

**Table:**

Refinement on *F*^2^	Primary atom site location: structure-invariant direct methods
Least-squares matrix: full	Secondary atom site location: difference Fourier map
*R*[*F*^2^ > 2σ(*F*^2^)] = 0.073	Hydrogen site location: inferred from neighbouring sites
*wR*(*F*^2^) = 0.170	H atoms treated by a mixture of independent and constrained refinement
*S* = 1.09	*w* = 1/[σ^2^(*F*_o_^2^) + (0.0648*P*)^2^ + 0.566*P*] where *P* = (*F*_o_^2^ + 2*F*_c_^2^)/3
2432 reflections	(Δ/σ)_max_ < 0.001
169 parameters	Δρ_max_ = 0.21 e Å^−^^3^
2 restraints	Δρ_min_ = −0.20 e Å^−^^3^

### Special details

**Table:**

Geometry. All s.u.'s (except the s.u. in the dihedral angle between two l.s. planes) are estimated using the full covariance matrix. The cell s.u.'s are taken into account individually in the estimation of s.u.'s in distances, angles and torsion angles; correlations between s.u.'s in cell parameters are only used when they are defined by crystal symmetry. An approximate (isotropic) treatment of cell s.u.'s is used for estimating s.u.'s involving l.s. planes.
Refinement. Refinement of *F*^2^ against ALL reflections. The weighted *R*-factor *wR* and goodness of fit *S* are based on *F*^2^, conventional *R*-factors *R* are based on *F*, with *F* set to zero for negative *F*^2^. The threshold expression of *F*^2^ > 2σ(*F*^2^) is used only for calculating *R*-factors(gt) *etc*. and is not relevant to the choice of reflections for refinement. *R*-factors based on *F*^2^ are statistically about twice as large as those based on *F*, and *R*- factors based on ALL data will be even larger.

### Fractional atomic coordinates and isotropic or equivalent isotropic displacement parameters (Å^2^)

**Table:**

	*x*	*y*	*z*	*U*_iso_*/*U*_eq_	
C1	0.3277 (2)	−0.2371 (7)	1.1123 (2)	0.0577 (10)	
H1A	0.2878	−0.2249	1.1550	0.087*	
H1B	0.3826	−0.1874	1.1447	0.087*	
H1C	0.3303	−0.4029	1.0914	0.087*	
C2	0.3509 (2)	−0.0767 (6)	0.9634 (2)	0.0428 (8)	
C3	0.4169 (2)	−0.2323 (6)	0.9573 (3)	0.0558 (10)	
H3	0.4305	−0.3561	1.0020	0.067*	
C4	0.4628 (2)	−0.2049 (7)	0.8849 (3)	0.0578 (10)	
H4	0.5075	−0.3099	0.8812	0.069*	
C5	0.4432 (2)	−0.0246 (6)	0.8182 (2)	0.0525 (9)	
H5	0.4745	−0.0076	0.7696	0.063*	
C6	0.3768 (2)	0.1321 (6)	0.8233 (2)	0.0468 (8)	
H6	0.3637	0.2548	0.7781	0.056*	
C7	0.3297 (2)	0.1078 (5)	0.8954 (2)	0.0378 (7)	
C8	0.21464 (19)	0.4042 (5)	0.8447 (2)	0.0365 (7)	
C9	0.14682 (19)	0.5534 (5)	0.8782 (2)	0.0356 (7)	
C10	0.0684 (2)	0.5852 (6)	0.8000 (2)	0.0516 (9)	
H10A	0.0671	0.4975	0.7406	0.062*	
H10B	0.0139	0.6022	0.8198	0.062*	
C11	0.1292 (2)	0.7886 (6)	0.8211 (2)	0.0500 (9)	
H11A	0.1116	0.9299	0.8537	0.060*	
H11B	0.1647	0.8252	0.7745	0.060*	
C12	0.13045 (19)	0.5525 (5)	0.9782 (2)	0.0366 (7)	
C13	0.0787 (2)	0.7537 (6)	1.0116 (3)	0.0559 (10)	
H13A	0.0740	0.7263	1.0770	0.084*	
H13B	0.0233	0.7553	0.9731	0.084*	
H13C	0.1059	0.9076	1.0059	0.084*	
N2	0.16185 (17)	0.3782 (4)	1.03227 (17)	0.0405 (7)	
O1	0.30181 (15)	−0.0837 (4)	1.03230 (16)	0.0589 (7)	
O2	0.22453 (15)	0.4164 (4)	0.76124 (15)	0.0523 (6)	
O3	0.14111 (17)	0.3914 (4)	1.12368 (16)	0.0580 (7)	
H3O	0.176 (2)	0.300 (7)	1.159 (3)	0.087*	
N1	0.26309 (17)	0.2616 (4)	0.90914 (18)	0.0392 (7)	
H1N	0.2470 (19)	0.254 (6)	0.9632 (16)	0.047*	

### Atomic displacement parameters (Å^2^)

**Table:**

	*U*^11^	*U*^22^	*U*^33^	*U*^12^	*U*^13^	*U*^23^
C1	0.067 (3)	0.060 (2)	0.048 (2)	0.008 (2)	0.0146 (18)	0.0174 (18)
C2	0.051 (2)	0.0407 (17)	0.0392 (18)	0.0018 (16)	0.0135 (15)	−0.0005 (15)
C3	0.061 (2)	0.050 (2)	0.057 (2)	0.0181 (19)	0.0139 (19)	0.0073 (17)
C4	0.058 (2)	0.056 (2)	0.064 (3)	0.0165 (19)	0.0211 (19)	−0.0074 (19)
C5	0.055 (2)	0.059 (2)	0.049 (2)	0.0000 (19)	0.0237 (17)	−0.0093 (18)
C6	0.054 (2)	0.0429 (18)	0.046 (2)	−0.0002 (17)	0.0155 (17)	−0.0008 (15)
C7	0.045 (2)	0.0327 (16)	0.0372 (17)	−0.0028 (15)	0.0115 (14)	−0.0061 (14)
C8	0.046 (2)	0.0311 (15)	0.0317 (17)	−0.0094 (15)	0.0058 (14)	−0.0012 (13)
C9	0.0413 (18)	0.0258 (14)	0.0394 (17)	−0.0050 (14)	0.0064 (14)	0.0022 (13)
C10	0.049 (2)	0.053 (2)	0.050 (2)	0.0036 (18)	0.0013 (16)	0.0030 (17)
C11	0.065 (2)	0.0338 (17)	0.051 (2)	0.0036 (17)	0.0092 (18)	0.0139 (15)
C12	0.0402 (19)	0.0257 (14)	0.0444 (18)	−0.0048 (14)	0.0089 (14)	−0.0011 (13)
C13	0.068 (3)	0.0383 (19)	0.067 (3)	0.0096 (18)	0.026 (2)	−0.0046 (17)
N2	0.0562 (18)	0.0345 (14)	0.0336 (14)	0.0018 (13)	0.0154 (12)	0.0017 (12)
O1	0.0641 (17)	0.0655 (16)	0.0524 (15)	0.0226 (13)	0.0245 (12)	0.0235 (12)
O2	0.0684 (16)	0.0570 (15)	0.0329 (13)	0.0035 (13)	0.0128 (11)	0.0033 (11)
O3	0.0794 (19)	0.0602 (16)	0.0402 (14)	0.0181 (14)	0.0264 (12)	0.0069 (11)
N1	0.0509 (17)	0.0354 (13)	0.0334 (15)	0.0070 (13)	0.0133 (13)	0.0018 (12)

### Geometric parameters (Å, °)

**Table:**

C1—O1	1.420 (4)	C8—C9	1.505 (4)
C1—H1A	0.9600	C9—C12	1.494 (4)
C1—H1B	0.9600	C9—C11	1.525 (4)
C1—H1C	0.9600	C9—C10	1.540 (4)
C2—O1	1.362 (4)	C10—C11	1.478 (5)
C2—C3	1.376 (4)	C10—H10A	0.9700
C2—C7	1.402 (4)	C10—H10B	0.9700
C3—C4	1.377 (5)	C11—H11A	0.9700
C3—H3	0.9300	C11—H11B	0.9700
C4—C5	1.369 (5)	C12—N2	1.275 (4)
C4—H4	0.9300	C12—C13	1.507 (4)
C5—C6	1.380 (5)	C13—H13A	0.9600
C5—H5	0.9300	C13—H13B	0.9600
C6—C7	1.385 (4)	C13—H13C	0.9600
C6—H6	0.9300	N2—O3	1.402 (3)
C7—N1	1.402 (4)	O3—H3O	0.85 (4)
C8—O2	1.229 (3)	N1—H1N	0.856 (17)
C8—N1	1.345 (4)		
			
O1—C1—H1A	109.5	C12—C9—C10	115.7 (3)
O1—C1—H1B	109.5	C8—C9—C10	112.2 (3)
H1A—C1—H1B	109.5	C11—C9—C10	57.7 (2)
O1—C1—H1C	109.5	C11—C10—C9	60.6 (2)
H1A—C1—H1C	109.5	C11—C10—H10A	117.7
H1B—C1—H1C	109.5	C9—C10—H10A	117.7
O1—C2—C3	125.3 (3)	C11—C10—H10B	117.7
O1—C2—C7	114.7 (3)	C9—C10—H10B	117.7
C3—C2—C7	120.0 (3)	H10A—C10—H10B	114.8
C2—C3—C4	120.0 (3)	C10—C11—C9	61.7 (2)
C2—C3—H3	120.0	C10—C11—H11A	117.6
C4—C3—H3	120.0	C9—C11—H11A	117.6
C5—C4—C3	120.7 (3)	C10—C11—H11B	117.6
C5—C4—H4	119.6	C9—C11—H11B	117.6
C3—C4—H4	119.6	H11A—C11—H11B	114.7
C4—C5—C6	119.9 (3)	N2—C12—C9	117.5 (3)
C4—C5—H5	120.0	N2—C12—C13	122.7 (3)
C6—C5—H5	120.0	C9—C12—C13	119.8 (3)
C5—C6—C7	120.4 (3)	C12—C13—H13A	109.5
C5—C6—H6	119.8	C12—C13—H13B	109.5
C7—C6—H6	119.8	H13A—C13—H13B	109.5
C6—C7—C2	119.0 (3)	C12—C13—H13C	109.5
C6—C7—N1	125.1 (3)	H13A—C13—H13C	109.5
C2—C7—N1	115.9 (3)	H13B—C13—H13C	109.5
O2—C8—N1	122.3 (3)	C12—N2—O3	112.9 (2)
O2—C8—C9	120.0 (3)	C2—O1—C1	118.0 (3)
N1—C8—C9	117.7 (2)	N2—O3—H3O	107 (3)
C12—C9—C8	123.9 (3)	C8—N1—C7	128.2 (3)
C12—C9—C11	117.6 (3)	C8—N1—H1N	114 (2)
C8—C9—C11	111.6 (3)	C7—N1—H1N	117 (2)
			
O1—C2—C3—C4	−179.1 (3)	C8—C9—C10—C11	−102.3 (3)
C7—C2—C3—C4	0.8 (5)	C12—C9—C11—C10	−104.3 (3)
C2—C3—C4—C5	−0.4 (6)	C8—C9—C11—C10	103.3 (3)
C3—C4—C5—C6	0.1 (6)	C8—C9—C12—N2	−16.4 (4)
C4—C5—C6—C7	−0.2 (5)	C11—C9—C12—N2	−165.1 (3)
C5—C6—C7—C2	0.6 (5)	C10—C9—C12—N2	129.6 (3)
C5—C6—C7—N1	177.7 (3)	C8—C9—C12—C13	163.4 (3)
O1—C2—C7—C6	179.0 (3)	C11—C9—C12—C13	14.7 (4)
C3—C2—C7—C6	−0.9 (5)	C10—C9—C12—C13	−50.6 (4)
O1—C2—C7—N1	1.7 (4)	C9—C12—N2—O3	−178.6 (2)
C3—C2—C7—N1	−178.2 (3)	C13—C12—N2—O3	1.7 (4)
O2—C8—C9—C12	−179.5 (3)	C3—C2—O1—C1	10.3 (5)
N1—C8—C9—C12	0.1 (4)	C7—C2—O1—C1	−169.6 (3)
O2—C8—C9—C11	−29.2 (4)	O2—C8—N1—C7	−0.4 (5)
N1—C8—C9—C11	150.4 (3)	C9—C8—N1—C7	−179.9 (3)
O2—C8—C9—C10	33.5 (4)	C6—C7—N1—C8	23.1 (5)
N1—C8—C9—C10	−146.9 (3)	C2—C7—N1—C8	−159.8 (3)
C12—C9—C10—C11	107.8 (3)		

### Hydrogen-bond geometry (Å, °)

**Table:**

*D*—H···*A*	*D*—H	H···*A*	*D*···*A*	*D*—H···*A*
N1—H1N···N2	0.86 (2)	1.94 (2)	2.670 (3)	142 (3)
O3—H3O···O2^i^	0.85 (4)	1.93 (2)	2.751 (3)	162 (4)

Symmetry codes: (i) *x*, −*y*+1/2, *z*+1/2.
